# Intimate partner violence among pregnant women in Rwanda

**DOI:** 10.1186/1472-6874-8-17

**Published:** 2008-10-10

**Authors:** Joseph Ntaganira, Adamson S Muula, Florence Masaisa, Fidens Dusabeyezu, Seter Siziya, Emmanuel Rudatsikira

**Affiliations:** 1 Department of Epidemiology, School of Public Health, National University of Rwanda, Kigali, Rwanda; 2 Department of Public Health, Division of Community Health, College of Medicine, University of Malawi, Blantyre, Malawi; 3 Department of Community Medicine, University of Zambia, Lusaka, Zambia; 4 Division of Epidemiology and Biostatistics, San Diego State University, San Diego, California, USA

## Abstract

**Background:**

Intimate partner violence (IPV), defined as actual or threatened physical, sexual, psychological, and emotional abuse by current or former partners is a global public health concern. The prevalence and determinants of intimate partner violence (IPV) against pregnant women has not been described in Rwanda. A study was conducted to identify variables associated with IPV among Rwandan pregnant women.

**Methods:**

A convenient sample of 600 pregnant women attending antenatal clinics were administered a questionnaire which included items on demographics, HIV status, IPV, and alcohol use by the male partner. Mean age and proportions of IPV in different groups were assessed. Odds of IPV were estimated using logistic regression analysis.

**Results:**

Of the 600 respondents, 35.1% reported IPV in the last 12 months. HIV+ pregnant women had higher rates of all forms of IVP violence than HIV- pregnant women: pulling hair (44.3% vs. 20.3%), slapping (32.0% vs. 15.3%), kicking with fists (36.3% vs. 19.7%), throwing to the ground and kicking with feet (23.3% vs. 12.7%), and burning with hot liquid (4.1% vs. 3.5%). HIV positive participants were more than twice likely to report physical IPV than those who were HIV negative (OR = 2.38; 95% CI [1.59, 3.57]). Other factors positively associated with physical IPV included sexual abuse before the age of 14 years (OR = 2.69; 95% CI [1.69, 4.29]), having an alcohol drinking male partner (OR = 4.10; 95% CI [2.48, 6.77] for occasional drinkers and OR = 3.37; 95% CI [2.05, 5.54] for heavy drinkers), and having a male partner with other sexual partners (OR = 1.53; 95% CI [1.15, 2.20]. Education was negatively associated with lifetime IPV.

**Conclusion:**

We have reported on prevalence of IPV violence among pregnant women attending antenatal care in Rwanda, Central Africa. We advocate that screening for IPV be an integral part of HIV and AIDS care, as well as routine antenatal care. Services for battered women should also be made available.

## Background

Intimate partner violence (IPV), defined as actual or threatened physical, sexual, psychological, and emotional abuse by current or former partners is a global public health concern affecting 25%–43% women in their lifetime [1-4]. The adverse health consequences of IPV have been documented in previous reports and include: mental disorders such as suicidal ideation, suicide and post-traumatic stress disorders; gynecological and obstetric disorders such as chronic pelvic pain and preterm deliveries; and infectious diseases such as HIV infection and other sexually transmitted infections [5-10].

One of the outcomes from the Fourth World Conference on Women held in Beijing in 1995 [11] was a call to encourage research in the causes and consequences of violence against women. The Conference also urged for the search of effective preventive measures and encouraged governments and non-governmental organizations to promote the delivery of public health interventions that will prevent and mitigate violence against women [11].

Although women suffer violence from many institutions and persons, the World Health Organization's "Multi-Country study on women's health and domestic violence against woman" confirms that the most common type of violence directed against women is actually carried out by their partners [12]. There are also data suggesting that pregnant women, when compared to the non-pregnant women, may be particularly at risk of IPV from their male partners.

In the United States, IPV occurring during pregnancy is a leading cause of maternal deaths [13]. This type of violence has also been associated with other adverse obstetric or neonatal outcomes such as with low birth weight and preterm delivery [9,14]. Both preterm and low-birth weight deliveries are a major source of infant mortality and long-term adverse health complications to children. The social and financial cost to families and society in general as a result of these outcomes is considerable.

In the developing world, data on IPV have largely been reported from the Demographic and Health Surveys (DHS), research projects conducted in South Africa [15-17]. The DHS reports also report the prevalence of violence directed at women in the general adult community. South Africa on the other hand is plagued as being a country with one of the highest prevalence of interpersonal violence in the world [2,18,19]. Male-to-female physical violence, including IPV, is particularly of concern in sub-Saharan Africa, not just because of the mental and physical consequences reported in other settings, but also of the association that violence against women is associated with HIV infection among affected women [20-22].

In order to contribute to the growing literature on IPV violence against women, and to explore the prevalence of the experience among pregnant women, we carried out the current study on women attending antenatal care in Rwanda. The aims of the study were: to estimate the prevalence of IPV among HIV infected and non-infected pregnant women and explore factors that may be associated with IPV among these women. We also specifically wanted to asses whether the odds of IPV differed among women HIV infected and those no infected.

## Methods

### Study population

In January 2006, trained research staff from the School of Public Health, National University of Rwanda administered a survey questionnaire to a convenient sample of 300 HIV(+) and 300 HIV (-) pregnant women attending prenatal care services in two urban antenatal clinics in Kigali and two rural antenatal clinics (one in South Province and another in North Province). Consecutive antenatal clinic attendees from urban (300) and rural (300) areas were recruited into the study.

The decision to recruit 300 participants in each arm of the study was reached as to get adequate sample size for HIV-positive versus HIV-negative; HIV status of the participants was known to the clinic staff. Using nQuery Advisor ^® ^software [23], with 300 study participants in each group, conducting a two-sided test and α = 0.05 and expecting an odds ratio of 1.3 (odds of IPV comparing HIV infected versus HIV uninfected) would enable the power of 75% [23]

In each clinic, there was a health education group session before the provision of the ANC services. Women were informed about the research during that session.

Participation was voluntary and women were informed that there will be no consequence for those who decide not to participate in the study; they will receive ANC services as usual. HIV+ pregnant women had a "liaison card" that allow clinic staff to identify them. The card was presented during the registration phase at the ANC clinic. ANC services were provided 3 days per week. We decided to enrol maximum 25 HIV-women per day in order to have variability in our sample. All HIV+ women present were enrolled as the prevalence was much lower. None of the recruited women refused to be interviewed

### Questionnaire and data collection

The questionnaire included items on demographics, intimate partner violence experience, alcohol use by male partner, and HIV status. To assess intimate partner violence exposure to intimate partner violence, women were asked: "Has any of your sex partners ever..." and "In the last twelve months, has your husband/partner: Threatened you verbally; Pulled your hair; Slapped you; Kicked you with fists; Threw you to the ground or kicked you with his feet; Choked you; Burned you or poured a hot liquid on you. A "Yes" to any of these options was coded as 1 versus 0 when none of these experiences were reported. These attributes have been identified to constitute IPV in previous studies in other settings [24-26]. Sexual abuse was defined as a report of forced sexual intercourse or intercourse without the will of the woman concerned.

A multiphase process was used to develop the research instrument to ensure that it was culturally and linguistically appropriate. The draft questionnaire in English was first translated into Kinyarwanda, the national language by two translators and was double checked. The instrument was refined after pre-testing with 50 pregnant women in one facility under the supervision of assistant lecturers of the Rwanda School of Public Health. A team of 10 trained data collectors under the direct supervision of the assistant lecturers conducted the fieldwork. Face-to-face interviews lasting 45 min-1 hour were conducted in a private room in order to ensure confidentiality. Before starting any interview, informed consent was sought and each woman respondent was informed of her right of not participating without penalty. A nurse counsellor was available to support victims of IPV.

### Statistical Methods

The measures used in this study were all based on items from the questionnaire. The demographic characteristics of interest were age, education (no formal education, elementary education, high school and beyond), marital status, sexual abuse before the age of 14 years, alcohol use by male partner, male partner having other wife or sexual partners, and HIV status. The choice of explanatory variables included in the study was based on the literature Conflict Tactics Scale [24] and have been used before by other authors with acceptable results [25-29]. The criterion for a variable to be an important confounder was a 15% change in OR when included in the model. Mean age and proportions of intimate partner violence in different groups were assessed. Both bivariate and multivariate odds of physical intimate partner violence were estimated using logistic regression analysis. All data analyses were done using SAS software 9.1 (SAS Institute Inc, Cary, North Carolina, United States).

### Human Subjects

The Institutional Review Board of the National University of Rwanda and the National Ethics Committee approved the study protocol. Informed verbal consent was obtained before administering the survey to study participants.

### Results

The mean age of the 600 participants was 30.2 years (SD: 5.9 years). Almost one five respondents (19.8%) reported having been sexually abused before the age of 14 years. About a third (35.1%) reported physical intimate partner violence in the last 12 months. (Table 1).

**Table 1:** Socio-demographic characteristics of the study population

Variable	Total (%) n	HIVpositive % (n)	HIVnegative % (n)
Age (years)			
18–25	26.5 (159)	28.0 (84)	18.7 (56)
26–34	50.2 (301)	52.3 (157)	48.0 (144)
35–47	23.3 (140)	19.7 (59)	33.3 (100)
Education			
No formal education	25.0 (150)	19.7 (59)	30.3 (91)
Elementary education	54.5 (325)	61.3 (184)	47.7 (143)
High school and beyond	20.5 (123	19.0 (57)	22.0 (66)
Marital status			
Married	46.3 (278)	43.3 (130)	49.3 (148)
Divorced	39.0 (234)	39.0 (152)	39.0 (152)
Single	14.7 (88)	17.7 (53)	11.7 (35)
Sexual abuse before 14 years of age			
Yes	19.8 (118)	23.2 (69)	16.4 (49)
No	80.2 (479)	76.9 (229)	83.6 (250)
Male partner's consumption of alcohol			
Never	23.5 (141)	19 (57)	28.0 (84)
Sometimes	39.7 (238)	43.3 (130)	36.0 (108)
Frequently or always	36.8 (221)	37.7 (113)	36.0 (108)
Male partner with other wife or sexual partners			
Yes	12.9 (76)	18.0 (53)	7.8 (23)
No	87.1 (513)	82.0 (241)	92.2 (272)
Physical violence in last 12 month	35.1 (210)	46.0 (138)	24.7 (74)

### Prevalence of intimate partner violence

Table 2 reports rates of intimate partner violence by HIV status. HIV+ pregnant women had higher rates of all forms of IVP violence than HIV- pregnant women: pulling hair (44.3% vs. 20.3%), slapping (32.0% vs. 15.3%), kicking with fists (36.3% vs. 19.7%), throwing to the ground and kicking with feet (23.3% vs. 12.7%), and burning with hot liquid (4.1% vs. 3.5%).

**Table 2:** Rates of intimate partner violence in the last 12 months among Rwandan pregnant women, 2006

Variable	Number of participants	% of total
		P < 0.001
Hair pulled	536	32.3
HIV+	248	44.3
HIV-	288	20.3
		P < 0.001
Thrown to the ground	578	18.0
HIV+	283	23.3
HIV-	295	12.7
		P < 0.001
Slapped	600	23.7
HIV+	300	32.0
HIV-	300	15.3
		P < 0.001
Kicked with fists or other object	553	28.0
HIV+	268	36.3
HIV-	285	19.7
		P = 0.137
Burned	579	3.8
HIV+	282	4.2
HIV-	297	3.5

Table 3 reports results from both bivariate and multivariate logistic regression analysis. In the bivariate analysis having formal education was negatively associated with lifetime IPV (OR = 0.30; 95% [0.17, 0.47] for elementary education, and OR = 0.18; 95% CI [0.10, 0.31] for high school or beyond]). HIV positive participants were more than twice likely to report physical IPV than those who were not HIV positive (OR = 2.60; 95% CI [1.84, 3.68]). Other factors positively associated with physical IPV included sexual abuse before the age of 14 years (OR = 2.82; 95% CI [1.87, 4.26]), having an alcohol drinking male partner (OR = 4.93; 95% CI [3.14, 7.72] for occasional drinkers and OR = 4.07; 95% CI [2.60, 6.38] for heavy drinkers), and having a male partner with other sexual partners (OR = 2.18; 95% CI [1.34, 3.55]. There was no difference in IPV between urban and rural areas. The direction of association was similar in the multivariate analysis as the bivariate results as shown in Table 1.

**Table 3:** Unadjusted and adjusted odds ratios with 95% confidence interval (CI) for factors associated with physical violence in the last 12 months among Rwandan pregnant women, 2006

Variable	Unadjusted OR [95% CI]	Adjusted OR [95% CI]
Age (years)		
18–25	1.00	1.00
26–34	1.13 [0.75, 1.70]	1.35 [0.84, 2.17]
35–47	0.70 [0.43, 1.09]	0.80 [0.48, 1.41]
Education		
No formal education	1.00	1.00
Elementary education	0.30 [0.17, 0.47]	0.35 [0.20, 0.60]
High school and beyond	0.18 [0.10, 0.31]	0.22 [0.12, 0.42]
Married status		
Married	1.00	
Divorced	0.85 [0.59, 1.23]	
Single	0.70 [0.42, 1.14]	
HIV status		
No	1.00	1.00
Yes	2.60 [1.84, 3.68]	2.38 [1.59, 3.57]
Sexual abuse before 14 years of age		
No	1.00	1.00
Yes	2.82 [1.87, 4.26]	2.69 [1.69, 4.29]
Male partner's consumption of alcohol		
Never	1.00	1.00
Occasional drinkers	4.93 [3.14, 7.72]	4.10 [2.48, 6.77]
Heavy drinkers	4.07 [2.60, 6.38]	3.37 [2.05, 5.54]
Male partner with other wife or sexual partners		
No	1.00	1.00
Yes	2.18 [1.34, 3.55]	1.53 [1.15, 2.20]

## Discussion

In a study of Rwandan women attending antenatal care at two government clinics, we report a 12 months prevalence of self-reported history of intimate partner physical violence. Life time prevalence was 35.3%. This estimate falls within the range of 25% to 43% reported elsewhere [1-4]. However, in these other settings, it has to be pointed out that these were life-time prevalence. This study also aimed to explore the association between HIV infection status and having experienced IPV in the past 12 months and life-time experience. We found that women who were HIV infected were more than 2 times likely to have experienced physical IPV compared to those not infected. These findings are in keeping with previous reported results from other settings [22]. As our study was a cross sectional in design, we cannot ascribe causation to any of the factors as being responsible for the outcome, such as that HIV was the cause of the violence.

We also found that women with no formal education were more likely to have experienced IPV than women with some education and above. Furthermore, previous history of sexual abuse, alcohol use by male partner and having a partner with other sexual partners were all independently associated with being a target of IPV. In a study by Paterson et al [29] in New Zealand, education was protective against IPV. Lack of education may result in lack of job opportunity or other income earning potential. In a study by Wilson et al [30] in North Carolina in the United States, despite the small sample size, all 25 women seeking care for IPV studied were unemployed.

In our study, we also found that women who reported that their male partners had other sexual partners were more likely to report exposure to IPV than those who did not report infidelity in their male partners. These findings are similar to what have been reported before by Karamagi et al in Uganda [22]. Van der Straten et al [31] have suggested that males may use violence to their partner's accusations of infidelity. Violent behaviors by male partner may also be a means to obtain sex against a woman's consent [32].

The role of alcohol use by male partners, like the other factors already examined above, is also likely to be complex. The dis-inhibition that may be associated with alcohol may result in a low threshold to violence. Alcohol use and household neglect that may result from such use may also facilitate development of marital or relationship tension that may result in violence. Alcohol use has also been reported to be associated with having multiple sexual partners [33-36], an issue that may also fuel couple discord. Furthermore, some persons may intentionally use alcohol in order to "hide" behind the alcohol in order to engage in antisocial behaviors such as violence against their partners. Gustafson [37] and Bushman [38] have reported the expectation that alcohol facilitate violent behaviors. Bushman [38] for example, has reported that research using real and mock alcoholic beverages shows that people who believe they have consumed alcohol begin to act more aggressively, regardless of whether they have consumed alcoholic beverage or not.

Sarkar [39] has reported that physical violence on pregnant women increases the risk for low birth weight infants, pre-term delivery and neonatal death and negatively affects breast-feeding postpartum. HIV itself results in similar maternal and neonatal outcomes. There is need to explore if there are joint effects of HIV and IPV on the maternal and child health outcomes.

### Limitations of the study

Our study has several limitations that need consideration. Firstly, this study recruited pregnant women who were attending antenatal care at government health clinics. To the extent that pregnant women differ from non-pregnant women in exposure to IPV, our findings may not be representative of all women in the study area. Stephenson et al [40] have reported that couples in which IPV occurs were unlikely to adopt modern family planning. This may suggest that abused women may be over-represented among pregnant women. On the other hand, although abused women may be more likely to become pregnant, it may not necessarily follow that these women will access antenatal care.

Jewkes [2] and Wilson et al [30] have reported that abused women experienced significant barriers to access healthcare. Male partners may prevent their female partners from accessing healthcare as a means of control [41]. This may suggest that these women may be under-represented in healthcare settings. Authors in previous research have also reported the increased occurrence of illness [42,43] among women exposed to IPV. These women may, therefore present more often to health care services than non-abused women with psychosomatic illnesses. For pregnant women in many setting, the antenatal clinic may be one such clinic. While we have explored all these possible scenarios, we do not have any data to substantiate the possibility of one mechanism that may have predominated against the other.

It is important to recognize that data were obtained through self-reports. There is possibility of inadvertent as well as intentional misreporting. We do not believe that women may have exaggerated the reports but rather that there may have been under-reporting of their experiences as has been observed in other settings [44,45]. As it was not possible to corroborate the women's reports with interview of male counterparts without endangering the women, we believe there is need to explore men's reports on IPV both as targets but more so as perpetrators as has been done elsewhere [46,47].

## Conclusion

We have reported on prevalence of physical intimate partner violence among pregnant women attending antenatal care in Rwanda, Central Africa. We find that IPV is common among pregnant women and that HIV infected women may be at greater exposure. We advocate that screening for IPV be an integral part of HIV and AIDS care, as well as routine antenatal care. We therefore call for a high index of suspicion of IPV among HIV infected women by prenatal care providers. We also suggest that counseling offered to women when testing for HIV should also consider screening for IPV and its associated factors. Furthermore, there should be a concerted effort to provide the necessary social, treatment and legal support for women who may avail themselves for such services. However, lack of availability of such services currently is an important shortfall that should be bridged.

## Competing interests

The authors declare that they have no competing interests.

## Authors' contributions

JN conceptualized the study, supervised data collection and participated in the interpretation of finding and drafting of manuscript. ASM: participated in the interpretation of finding and drafting of manuscript. FM: participated in study design and participated in the interpretation of findings and drafting of manuscript. FD: participated in study design and participated in the interpretation of findings and drafting of manuscript. SS: participated in the interpretation of findings and drafting of manuscript. ER: conducted data analysis and participated in the interpretation of findings and drafting of manuscript. All the authors approved the manuscript.

## Pre-publication history

The pre-publication history for this paper can be accessed here:


